# Deep Brain Stimulation of the Medial Septum Improves Okadaic Acid‐Induced Impairment of Spatial Working Memory and Restores the Cholinergic Activity in the Hippocampus

**DOI:** 10.1002/alz.087892

**Published:** 2025-01-03

**Authors:** MARIAM CHIGHLADZE, Khatuna Rusadze, Maia Burjanadze, Manana Dashniani, Lali Kruashvili

**Affiliations:** ^1^ Ivane Beritashvili Center of Experimental Biomedicine, Tbilisi Georgia; ^2^ Tbilisi State University, Tbilisi Georgia

## Abstract

**Background:**

There is growing evidence from laboratory and clinical trials that deep brain stimulation (DBS) at memory associated structures enhances cognitive functions. Best site for memory enhancing‐DBS is still unclear. The medial septum (MS), the important modulator of the hippocampal neural network, might be a key target to accomplish therapeutic efficacy in memory impaired patients.

In the present study, we evaluated the effect of medial septum (MS) deep brain stimulation (DBS) on okadaic acid (OA) induced impairment of spatial working memory and neuropathological changes in the hippocampus in rats.

**Method:**

Rats were divided in four groups: rats in the normal group had no surgical procedure (group – N); rats in the lesion group had ICV administration of OA (group – O); rats in the implantation group had ICV administration of OA and implantation of an electrode in the MS (group – O/I); rats in the stimulation group had ICV administration of OA and electrical stimulation of the MS (group – O/S). In the chronic DBS experiment, animals received stimulation (60 Hz, 60 𝜇s, 50 𝜇A) 2 hr daily for a period of 2 weeks. In behavioral experiments the possible beneficial effect of chronic DBS of MS on the OA‐induced spatial short‐term memory impairment was examined in spontaneous alternation task in the plus‐maze. At the end of the behavioral experiments, six rats from each group were used by random sampling in the histological studies

**Result:**

The results showed that MS DBS improves OA‐induced impairment of spatial working memory and restores acetylcholinesterase (AChE) immune‐stained cells numbers in the hippocampal CA1 and CA3 fields. We hypothesize that the enhancement of working memory function may be associated with an increase in hippocampal neurogenesis and hippocampal cholinergic activity induced by MS DBS in a rat model of AD.

**Conclusion:**

we suggest that the enhancement of working memory function may be associated with an increase in hippocampal neurogenesis and recovery of the AChE immune‐stained cells numbers in the hippocampus induced by MS DBS in a rat model of AD. These results allow us to identify the medial septum as one of the stimulation sites for improving memory function.

